# Big Area Additive Manufacturing of High Performance Bonded NdFeB Magnets

**DOI:** 10.1038/srep36212

**Published:** 2016-10-31

**Authors:** Ling Li, Angelica Tirado, I. C. Nlebedim, Orlando Rios, Brian Post, Vlastimil Kunc, R. R. Lowden, Edgar Lara-Curzio, Robert Fredette, John Ormerod, Thomas A. Lograsso, M. Parans Paranthaman

**Affiliations:** 1 Oak Ridge National Laboratory, Oak Ridge, TN 37831, USA; 2 Ames Laboratory, Ames, Iowa 50011, USA; 3 Magnet Applications, Inc., DuBois, PA 15801, USA

## Abstract

Additive manufacturing allows for the production of complex parts with minimum material waste, offering an effective technique for fabricating permanent magnets which frequently involve critical rare earth elements. In this report, we demonstrate a novel method - Big Area Additive Manufacturing (BAAM) - to fabricate isotropic near-net-shape NdFeB bonded magnets with magnetic and mechanical properties comparable or better than those of traditional injection molded magnets. The starting polymer magnet composite pellets consist of 65 vol% isotropic NdFeB powder and 35 vol% polyamide (Nylon-12). The density of the final BAAM magnet product reached 4.8 g/cm^3^, and the room temperature magnetic properties are: intrinsic coercivity *H*_*ci*_ = 688.4 kA/m, remanence *B*_*r*_ = 0.51 T, and energy product (*BH*)_max_ = 43.49 kJ/m^3^ (5.47 MGOe). In addition, tensile tests performed on four dog-bone shaped specimens yielded an average ultimate tensile strength of 6.60 MPa and an average failure strain of 4.18%. Scanning electron microscopy images of the fracture surfaces indicate that the failure is primarily related to the debonding of the magnetic particles from the polymer binder. The present method significantly simplifies manufacturing of near-net-shape bonded magnets, enables efficient use of rare earth elements thus contributing towards enriching the supply of critical materials.

NdFeB permanent magnets are frequently classified into sintered and bonded magnets^1^. While sintered magnets retain full density and offer high energy product, bonded magnets have high degree of net-shape formability and intermediate energy product. Bonded permanent magnets are fabricated by blending magnetic powders with a polymer as binder, and then molded into desired shapes utilizing several commercial processing methods including injection molding, compression molding, extrusion, and calendering, see details in ref. 2. Recently, bonded permanent magnets have experienced accelerated industrial applications due to their advantages such as intricate shapes, low weight and cost, superior mechanical properties and corrosion resistance, etc. ref. 2 Nd_2_Fe_14_B was first discovered as a strong permanent magnet in 1984^3^,^4^. It adopts a tetragonal crystal structure (P4_2_/mnm) with the easy magnetic axis along the c axis^5^. It possesses high magnetic energy product as large as 512 kJ/m^3^ (64 MGOe), with a Curie temperature *Tc* = 585 K and a high magnetic anisotropy constant *K*_*1*_ of 4.5 MJ/m^3^ arising from the strong spin-orbit coupling in Nd^6^. In fact, developing better NdFeB bonded magnets has been heavily researched^7^,^8^,^9^,^10^,^11^. Magnet powder properties, processing temperature, loading factor, magnet density and degree of orientation are critical process variables for improving magnetic and mechanical properties of NdFeB bonded magnets^12^.

Nevertheless, the conventional techniques used for bonded magnets fabrication have several drawbacks such as specific tooling requirement for each design and limitations in shape flexibility and complexity. Additive Manufacturing (AM) is an emerging technology that builds three dimensional objects from computer-aided design (CAD) models by adding layer-by-layer of material^13^. It has attracted tremendous attention from both the research^14^,^15^ and industrial communities. The prevailing industrial applications are due to the advantages over conventional subtractive manufacturing processes such as: minimum materials waste (if any) and energy usage, less process time, no additional tooling costs, no size and shape limitations. Since permanent magnets are frequently composed of rare earth elements, most of which are defined as critical materials, AM could potentially offer an effective way to reduce the usage of critical materials during bonded magnets fabrication. Very recently, Huber *et al*.^15^ used an end-user 3D printer to fabricate isotropic NdFeB bonded magnets. The density of the printed part is 3.57 g/cm^3^, which is lower than that of the injection molded magnets (4.35 g/cm^3^), leading to reduced remanence. Binder jetting AM technique has been employed by our group to manufacture Nd-Fe-B bonded magnets^16^. However, the hard magnetic properties of the previous magnet product are not satisfactory due to the technique’s limitation in producing sufficiently dense parts. In this work, we utilized the Big Area Additive Manufacturing (BAAM) system located at the Manufacturing Demonstration Facility at Oak Ridge National Laboratory to fabricate near-net-shape isotropic NdFeB bonded magnets. The BAAM system deposits high-performance engineered thermoplastics and customized thermoplastic composites via melt extrusion processing, which enables rapid manufacturing of parts completely unbounded in size^17^. Figure 1(a) shows the BAAM printing process of the bonded magnets: the nozzle deposits layers of magnetic materials which are fused together and solidify to form the desired shape. Instead of requiring pre-extruded filament feedstock commonly used in industry standard extrusion-based system, BAAM combines melting, compounding, and extruding functions to deposit polymer product at a controlled rate, a schematic is shown in Fig. 1(b). The feedstock materials here are magnetic pellets composed of 65 vol% isotropic NdFeB powder (MQP-B+-10118-070) and 35 vol% Nylon-12. It is worth mentioning that the printing of the extruded nylon magnet composite flows even better than the widely explored 3D printing plastic filament acrylonitrile butadiene styrene (ABS), and renders high accuracy. The magnetic, mechanical, and microstructural properties of the BAAM fabricated bonded magnets are investigated and compared with respect to the traditional injection molded commercial products made from the same starting materials. The results obtained with the BAAM fabricated bonded magnets are much better than those of traditional injection molded magnets.

## Results and Discussion

### Magnetic Properties

The room temperature magnetization data obtained on the BAAM and injection molded bonded magnets are shown in Fig. 2(a,b). The injection molded magnets were produced by Magnet Applications Inc. with the Nd-Fe-B powder (MQP-B+-10118-070 isotropic) mixed with 35% volume fraction of Nylon-12 binder. Figure 2(a) shows the de-magnetization curves for BAAM and IM magnets. The density of the BAAM and injection molded magnets are 4.8 g/cm^3^ and 4.9 g/cm^3^ respectively. It can be seen that both magnets retain the hard magnetic behavior of the original pellets. The magnetic properties of the BAAM magnet do not show any noticeable degradation compared to those of the starting pellets (see Figure S4 in the Supporting Information). Moreover, the BAAM magnet shows a slightly better hysteresis loop shape in the second quadrant of the demagnetization plot, and higher *H*_*ci*_ and *B*_*r*_ compared to the injection molded magnets. The BAAM magnet has the intrinsic coercivity *H*_*ci*_ = 688.4 kA/m, remanence *B*_*r*_ = 0.51 T, and saturation magnetization 4
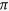
*M*_*s*_ ≈ 0.74 T.

The energy product (*BH*)_max_ quantifies the magnetostatic energy a permanent magnet material can store therefore characterizes how strong the magnet is. Recently a large amount of magnet research has been concentrated on enhancing (*BH*)_max_ through microstructure engineering^18^, exchange coupling through hybridizing hard/soft magnets (e.g. Nd_2_Fe_14_B/αFe)^19^, etc. The ideal M-H hysteresis loops for a permanent magnet should be square shaped, which gives a (*BH*)_max_ = *¼ μ*_*0*_*J*_*s*_^2^, where *J*_*s*_ = *4πM*_*s*_ is the saturation magnetization in unit of gauss^20^. The M-H loops of real permanent magnet materials deviate from ideal shape, as shown in Fig. 2(a), which results in a maximum achievable (*BH*)_max_ lower than (*BH*)_max_ = *¼ μ*_*0*_*J*_*s*_^2^. In fact, a balance between *H*_*ci*_, *B*_*r*_, and demagnetization curve squareness is imperative to obtain high energy product. Figure 2(b) presents the (*BH*) vs. H (−400 to 0 kA/m) for the BAAM and injection molded Nd-Fe-B magnets, whereby (*BH*)_max_ was determined as the maximum point in the plot. (*BH*)_max_ = 43.49 kJ/m^3^(5.47 MGOe) and 36.17 kJ/m^3^ (4.55 MGOe) are obtained for BAAM and IM magnets respectively. In real applications, the magnets are frequently exposed to elevated temperatures. Therefore, we investigated the magnetic properties of the BAAM fabricated magnets in the temperature interval of 300 K to 400 K. Figure 2(c) shows the second quadrant de-magnetization curves of BAAM printed magnet measured from 300 K to 400 K. Figure 2(d) shows the variation of the energy product of the BAAM magnet with increasing temperature. Magnetization data measured at various temperatures for IM magnets are plotted in Figure S2 in the Supporting Information. All the magnetic characteristics for both the BAAM and injection molding fabricated magnets are summarized in Table 1. It can be clearly seen that all the magnetic parameters decrease with increasing temperature, which can be readily understood as the increased thermal energy will disturb the alignment of the spins. The decreasing rates for BAAM magnet are 0.34%/K, 0.11%/K and 0.26%/K for *H*_*ci*_, *B*_*r*_, and (*BH*)_max_ respectively. Note that the coercivity is the magnetic field when *B* = 0 whereas the intrinsic coercivity is the magnetic field when *M* = 0.

### Flux Aging Loss

Figure 3(a,b) present the flux aging loss of BAAM magnet. The flux loss (%) evaluates the environmental stability of the magnet, and is defined as (*B*_*f*_ − *B*_*i*_)/*B*_*i*_ × 100%, where *B*_*f*_ and *B*_*i*_ are the flux density after certain duration of elevated temperature exposure and initial value respectively. Rectangular shaped magnet specimens with approximate dimensions of 30 mm × 15 mm × 10 mm were used for the flux measurements. The permeance coefficients (Pc) of the specimens are approximately 0.9. Figure 3(a) shows the flux loss with time for three different temperatures for up to 1000 h. Figure 3(b) shows the flux loss for BAAM magnet aged at various temperatures for 200 h. This plot is usually used to determine the maximum operation temperature of a magnet, *e*.*g*., the aging temperature below which the magnet exhibits a flux loss below certain value (5% or 10%). The flux losses after 200 hours exposure at 350 K, 400 K, and 450 K are 2.3%, 7.1%, and 13.3% respectively, which are comparable to the flux aging loss values of the compression molded magnets made from MQP-B+ powder^1^. The Magnetic flux loss is primarily related to spin relaxation and corrosion/oxidation^21^. A high coercivity is usually required to overcome the spin relaxation at elevated temperatures. It has been found that bonded magnets prepared from microdispersion coated powders exhibit improvement in flux aging loss^21^. Hence, it is essential to optimize the starting powders in order to improve the thermal stability of the final bonded magnets.

### Microstructure

Figure 4(a,b) show the morphologies of the starting nylon magnet composite pellets used for printing and the BAAM fabricated bonded magnet respectively. The plate-shaped magnetic particles (bright), which have sizes in the range of 20–200 μm are separated by nylon polymer binder (dark). It can be observed that the magnetic particles in the printed magnet are preferentially aligned, possibly due to melting and extrusion during printing. Each particle of MQP powder is isotropic and made up of many submicron grains. So while the alignment may influence mechanical properties compared to injection molded magnets, however, it should not influence the magnetic properties in terms of *B*_*r*_. It is possible that the slight increase in *H*_*ci*_ for BAAM magnets compared to IM magnets, as observed in Fig. 2(a), is due to the presence of shape anisotropy (Fig. 4(b)).

### Mechanical Properties

Polyphenylene-sulfide (PPS) and polyamide (Nylon) are the two commonly used polymer binders for bonded magnets production. It has been found that PPS based magnets usually exhibit higher ultimate strengths, higher temperature operation but lower ductility than Nylon based magnets^22^. The Nylon polymer used in this work is a Nylon-12, which is a thermoplastic binder with a melting point of 177 °C. This type of binder usually offers the material superior mechanical flexibility and improves corrosion resistance^2^. Figure 5 presents the room temperature tensile stress-strain curves of four BAAM fabricated Nd-Fe-B magnets. Four dog-bone shaped specimens were tested in order to determine the degree of variability in microstructure and mechanical properties between samples. Note that the “tails” at the end of the curve in Fig. 5 are associated with the test conditions. Specimen 4 (blue curve) was not unloaded in a controlled manner as in the other three specimens. It can be seen that the materials exhibited a well-defined linear regime followed by ductile behavior before failure. The inset to Fig. 5 shows images of the four specimens after tensile testing indicating the location of failure. The BAAM magnets have an average Young’s modulus of 4.29 GPa, ultimate tensile strength of 6.60 MPa, and ultimate strain of 4.18%. Details for each sample are given in Table 2. The small standard deviation for all the three characteristics indicates good uniformity between samples. For comparison, Nylon-11 based injection molded NdFeB magnets made with 62 vol % spherical powders were found to exhibit similar mechanical properties with an ultimate tensile strength of 5 MPa, and an ultimate strain of 4.6%^23^. It should be noted that the mechanical strength of NdFeB bonded magnets significantly depends on the magnetic powder loading fraction and also the shape of the powder. Garrell *et al*.^23^ has shown that magnets made of irregular melt-spun powders exhibited higher tensile strength compared to those made from atomized spherical powders.

To investigate the failure mechanism, the fracture surfaces after tensile tests were examined with SEM, as shown in Fig. 4(c). Areas marked by red circles are where the magnetic particles were pulled out during the tensile test, which indicates that the failure is largely due to the debonding between the magnetic particles and the nylon binder. Note that similar failure mechanism has been reported previously for injection molded NdFeB magnets^23^.

### Summary

Bonded magnets offer a better option for making low cost intricate shapes from isotropic powder when the high magnetic performance of sintered magnets is not required. Injection molding is a well-established method for fabricating complex shaped bonded magnets. Here we propose a novel alternate approach - Big Area Additive Manufacturing (BAAM) - to fabricate near-net-shape isotropic NdFeB bonded magnets. Magnetic and mechanical characterizations demonstrate that the BAAM fabricated magnets can compete with or outperform the injection molded magnets. In addition, additive manufacturing offers significant advantages such as cost effectiveness (no tooling required), fast speed (simple procedure), and capability of producing parts of unlimited in sizes and shapes. Therefore, BAAM provides an effective method in realizing arbitrary shape with minimum cost and waste, and has the potential to revolutionize large-scale industry production of bonded magnets. In the future work, the effect of binder type, loading fraction of the magnetic powder, anisotropic particles, and processing temperature on the magnetic and mechanical properties of the printed bonded magnets will be investigated.

## Methods

### Materials Preparation

The starting nylon magnet composite pellets with 65 vol% Nd-Fe-B powder (MQP-B+-10118-070 isotropic) and 35 vol% Nylon-12 binder were prepared by Magnet Applications Inc. MQP-B+ powder (pre-milled to 80 mesh) and Nylon-12 in desired ratios were blended to a uniform mixture, which was fed into a pre-feed hopper with a powered auger on the outlet that feeds directly into the throat of the twin screw barrels. The feed rate was precisely controlled to be 10 kg/hour to obtain uniform pellets. As the blended material traveled through the barrel on the twin screw compounder the four zones were temperature controlled with the rear zone being the coolest ~195 °C and the front zone being the highest ~210 °C. The die is typically set at a slightly lower temperature than the front zone (~200 °C) to help begin the cooling process. The material flows through the barrel and is pushed through a multi orifice die with ~1/8″ holes while the die face cutter rotates to slice the pellets. The speed of the cutter controls the length of the pellet to achieve uniform length to O.D. pellet, in this case 1/8″ × 1/8″. Forced air is blown on the pellets to avoid bonding. The pellets were further dried at 60 °C for 4 hours, and then used as starting feedstock materials for the BAAM process. The characteristics of the Nd-Fe-B powders used are: Curie temperature *Tc* = 360 °C, intrinsic coercivity *H*_*ci*_ = 716–836 kA/m, energy product (*BH*)_max_ ~ 127 kJ/m^3^, and theoretical density ρ = 7.64 g/cm^3^.

**Figure 1:**
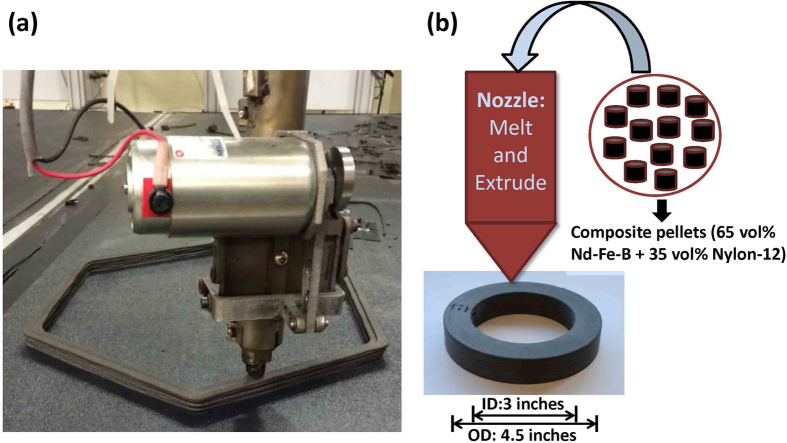
Schematics. (**a**) Image of the nozzle depositing layers of magnetic materials on the print bed; (**b**) Schematic of the melt and extrude process, right underneath the nozzle is a printed magnet in a hollow cylinder shape with an OD × ID of ~4.5 inch × 3 inch.

**Figure 2:**
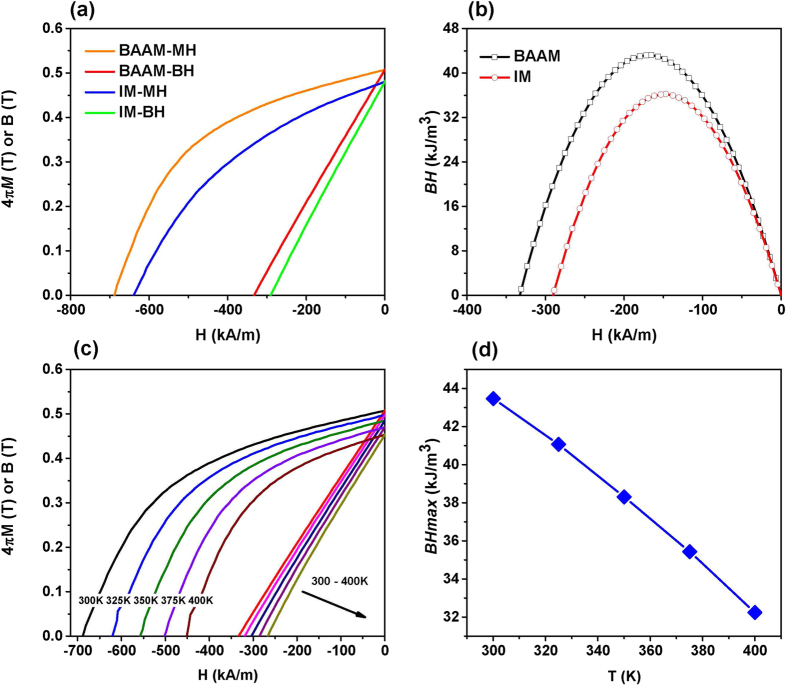
Magnetic properties of Big Area Additive Manufacturing (BAAM) and Injection Molding (IM) fabricated NdFeB bonded magnets (**a**) room temperature de-magnetization curves (MH and BH) for both BAAM and IM magnets; (**b**) room temperature maximum energy product for BAAM and IM magnets. (**c**) De-magnetization curves for BAAM magnets measured at elevated temperatures from 300 K to 400 K. (**d**) Maximum energy product as a function of temperature for BAAM magnet. Note that there are two sets of units in magnetism: SI and CGS. Conversion for some frequently used units are: 10 kG = 1 T; 1 Oe = 79.6 A/m; 1 MGOe = 7.95 kJ/m^3^, and B (G) = H (Oe) + 4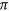 M (emu/cm^3^)^25^.

**Figure 3:**
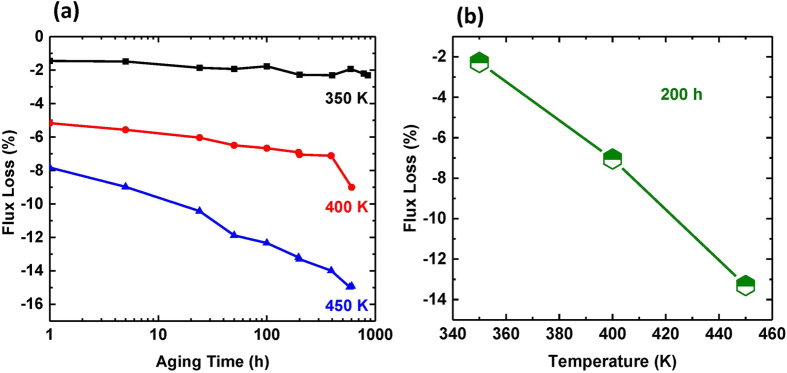
Thermal stability of the BAAM magnets. Flux aging loss for BAAM magnet as a function of (**a**) Aging Time (0–1000 h); (**b**) Temperature (350 K, 400 K, and 450 K) after 200 hours of exposure.

**Figure 4:**
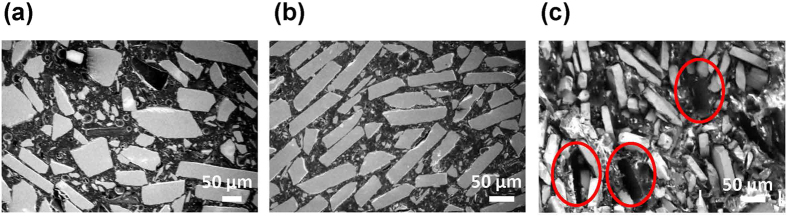
SEM micrographs (**a**) the starting composite pellets; (**b**) the BAAM printed bonded magnets; (**c**) the fractured surface of the BAAM magnets after tensile testing.

**Figure 5:**
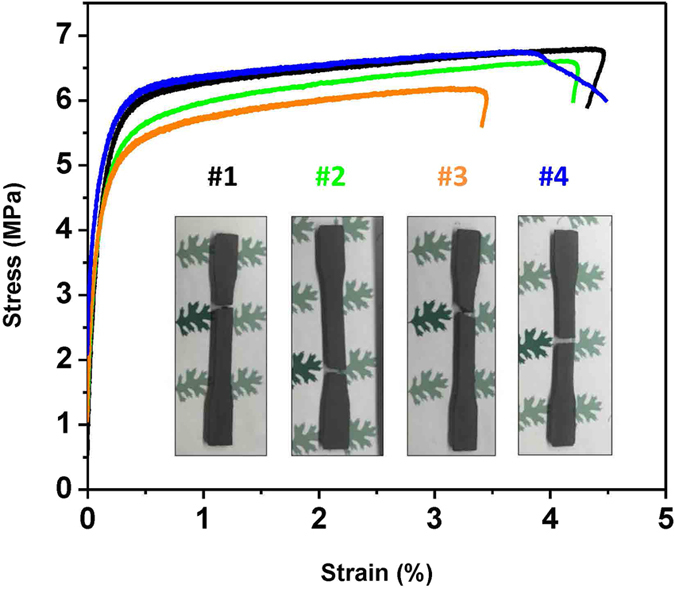
Mechanical properties of the BAAM fabricated NdFeB magnets. Tensile stress-strain curves of the BAAM fabricated Nd-Fe-B magnets; the inset shows the images of the four samples after tensile testing indicating the location of failure.

Three dimensional printing was performed at Manufacturing Demonstration Facility at Oak Ridge National Laboratory with the Big Area Additive Manufacturing (BAAM) system with a build volume of 3.56 m × 1.65 m × 0.86 m. The main components of the BAAM include the gantry system, single screw extruder, and a heated bed. The gantry uses linear drive motors to position the extruder with ± 0.0254 mm accuracy and was operated at a constant velocity of 25.4 mm/s during the printing process. The extruder is 25 mm in diameter with an aspect ratio of 12 (i.e. length/diameter = 12) and is used to melt nylon NdFeB composite pellets and deposit molten materials at a rate consistent with the gantry movement and desired bead profile. Details of the BAAM process is described in refs 17 and 24. The temperature at the orifice exit of the extruder was approximately 270 °C. The first layer was deposited on an acrylonitrile butadiene styrene (ABS) sheet that was placed on top of the heated aluminum table kept at a constant temperature of 90 °C. A hollow cylinder shape with an OD × ID of 4.5″ × 3″ of NdFeB bonded magnets (as shown in Fig. 1(b)) were printed. In addition, a large hexagon magnet sample (as shown in Fig. 1(a)) was also printed in order to measure the mechanical properties.

### Characterization

The magnetization data were obtained at 300–400 K with a vibrating sample magnetometer up to a maximum applied field of 3 T. Thermal transitions of the composite pellets were determined with a differential scanning calorimeter (DSC), which indicated that the starting composite pellets melt at 220 °C (see Figure S1 in the Supporting Information). Flux measurements were carried out using Helmholtz Coils with a fluxmeter (Model 2130) from Magnetic Instrumentation Inc. The samples were magnetized at 9 T and were exposed to elevated temperatures in furnaces for various periods of time. Each flux measurement was repeated for at least ten times, and the average value was used. Morphologies of the starting pellets, printed magnets and fracture surfaces were examined by Scanning Electron Microscopy (Hitachi S-4700). Cross sections of the specimens were polished and Au coated with an SPI Module Sputter Coater (12150-AB). The magnet samples printed were machined to a dog-bone shape for tensile evaluation according to an ASTM D638 Type I standard. Tensile testing was carried out at ambient conditions using a servohydraulic testing machine at a constant crosshead displacement rate of 7.6 μm/second. An extensometer with a 25.4-mm gauge length was used to measure axial strain. A slight pre-load of about 1.5 MPa was applied to the test specimens prior to the start of the test.

## Additional Information

**How to cite this article**: Li, L. *et al*. Big Area Additive Manufacturing of High Performance Bonded NdFeB Magnets. *Sci. Rep*. **6**, 36212; doi: 10.1038/srep36212 (2016).

**Publisher’s note**: Springer Nature remains neutral with regard to jurisdictional claims in published maps and institutional affiliations.

## Figures and Tables

**Table 1:** Magnetic properties of BAAM and IM fabricated bonded NdFeB magnets measured at various temperatures (300 K to 400 K).

Sample	Temperature (K)	*H*_*ci*_ (kA/m)	*H*_*c*_ (kA/m)	*B*_*r*_ (T)	(*BH*)_max_ (kJ/m^3^)	*4πM*_*3T*_ (T)
BAAM	300.00	688.37	357.31	0.51	43.49	0.74
	325.00	620.72	342.19	0.50	41.02	0.74
	350.00	557.06	323.89	0.48	38.32	0.73
	375.00	502.15	303.99	0.47	35.46	0.73
	400.00	452.01	281.71	0.45	32.20	0.72
IM	300.00	639.82	289.67	0.48	36.17	0.75
	325.00	577.75	274.55	0.47	33.47	0.74
	350.00	525.23	258.63	0.45	30.61	0.73
	375.00	479.87	243.51	0.43	27.59	0.72
	400.00	436.89	226.80	0.41	24.65	0.70

**Table 2:** Mechanical properties of BAAM fabricated bonded NdFeB magnets measured at room temperature.

	Modulus [GPa]	Ultimate Tensile Strength [MPa]	Ultimate Strain [%]
#1	4.53	6.81	4.43
#2	4.42	6.62	4.23
#3	4.48	6.20	3.45
#4	3.74	6.77	4.62
Average	4.29	6.60	4.18
Std. Dev	0.37	0.28	0.51
